# Frequency of depression, anxiety and stress among the undergraduate physiotherapy students

**DOI:** 10.12669/pjms.342.12298

**Published:** 2018

**Authors:** Annosha Syed, Syed Shazad Ali, Muhammad Khan

**Affiliations:** 1 Annosha Syed, PP-DPT Student, Institute of Physical Medical & Rehabilitation, Dow University of Health Sciences, Karachi, Pakistan; 2 Syed Shazad Ali, Assistant Professor, Institute of Physical Medical & Rehabilitation, Dow University of Health Sciences, Karachi, Pakistan; 3 Muhammad Khan, Director, Department of Physiotherapy & Rehabilitation, Lady Reading Hospital, Peshawar, Pakistan

**Keywords:** KEY WORDS: Anxiety, Stress, DASS-42, Depression, Physiotherapy

## Abstract

**Objectives:**

To assess the frequency of Depression, Anxiety and Stress (DAS) among the undergraduate physiotherapy students.

**Methods:**

A descriptive cross sectional study was conducted in various Physiotherapy Institutes in Sindh, Pakistan among undergraduate physiotherapy students. The total duration of this study was 4 months from September, 2016 to January, 2017. Data was collected from 267 students with no physical and mental illness; more than half were female students 75.3%. They were selected through Non probability purposive sampling technique. A self-administered standardized DASS (depression, anxiety and stress scale) was used to collect data and result was analyzed using its severity rating index. Data was entered and analyzed by using SPSS version 21. Descriptive statistics including the frequency of depression, anxiety, stress and demographic characteristic of the participant was collected.

**Results:**

The mean age of students was 19.3371±1.18839 years. The Frequency of depression, anxiety and stress found among undergraduates Physiotherapy students was 48.0%, 68.54% and 53.2%, respectively.

**Conclusions:**

It was observed that the frequency of depression, anxiety and stress among physiotherapy undergraduates students were high. It suggests the urgent need of carrying out evidence based Psychological health promotion for undergraduate Physiotherapy students to control this growing problem.

## INTRODUCTION

Psychological morbidities are most common psychiatric health problem worldwide.^1^ Mental health among university undergraduate students represents as important and developing public health concern. Depression is extremely dominant and widespread problem across the nation and predicted to be the leading cause of disease burden by 2030.^2^-^4^ According to WHO, depression is a common mental disorder, characterized by sadness, loss of interest or pleasure, feelings of guilt or low self-worth, disturbed sleep or appetite, low energy and poor concentration.^2^

Anxiety is considered as a state of uneasiness, it’s a bodily response to a perceived danger that could be real or imaginary and triggered by an individual’s thoughts, beliefs and feelings.^3^ Generally, academic stresses develop the sense of competition and motivation among students and encourage learning. However, sometimes this stress produces anxiety and feelings of helplessness, leading to stress-related disorders and adversely affecting academic and non-academic performance.^5^

Students with DAS are prone to academic difficulties, drop outs, relationship disturbance with friends and family members, failure to cope with anxious situation, and it could advance to panic disorders.^2^,^6^,^7^ That leads to lack of self-confidence, compromises the ability to cope with daily life problems which directly affects the academic performance of student.^8^,^9^ This increased prevalence can represent the fact that university students are supposed to prepare for their professional careers along with the increased academic and social responsibilities which leads to a numerous factor resulting in depression, anxiety and stress. The prevalence varies from country to country as well as from institution to institution.^1^ Currently, mental health morbidities is an important public health problem and it is a leading cause of disability worldwide accounting for one third of disability adjusted life years (DALYs).^10^,^11^

A study among undergraduate students in Canada showed that 30% of students has psychological morbidities.^12^ In addition, over 50% students in United State,^13^ 53% of students in Australia^14^ and 41.9% of students in Malaysia^15^ experienced mental health problems. In Turkey, depression, anxiety and stress were recorded as 27.1%, 47.1% and 27% respectively.^16^ Dahlin et al. reported the prevalence of stress to be as high as 12.9%.^17^ Another study has shown that Academics and taking exams are the most powerful stresses in medical and paramedical students.^18^ Rosenthal and Okie in 2005 reported higher prevalence of psychological problems such as stress, anxiety, and depression among medical students than in the general population and age matched peers.^19^

Asia seems to be suffering more for DAS. A study conducted on 353 medical university students in India reported that more than half of the respondents were affected by depression (51.3%), anxiety (66.9%) and stress (53%).^20^ In 2013, studies conducted in Iran reported that 38% depression among university students.^4^

Earlier number of studies conducted on DAS in undergraduate students in Pakistan, a study on undergraduate engineering student reported significant (73.8%) level of anxiety and depression.^2^ Another study reported very high prevalence of anxiety and depression (70%) among medical students in Pakistan.^21^ a study on medical students reported high level of anxiety in comparison to depression and stress.^22^ Similarly, studies conducted on medical students reported that moderate level of anxiety(43.89% ) and depression (60%).^23^,^24^

To authors knowledge none of the study has reported the prevalence depression, anxiety and stress among physiotherapy undergraduate students in Sindh Pakistan. Therefore, the purpose of this study was to estimate the prevalence of depression, anxiety and stress among undergraduate physiotherapy students.

## METHODS

A descriptive observational study was conducted among undergraduate physiotherapy students. Students were selected from various Institutes located in different cities of Sindh, Pakistan. The total duration of this study was four months from September 5, 2016 to January 5, 2017. A total 267 participant was included in the study. The sample size was calculated on the basis of prevalence of 51%^20^ as mentioned in the study and utilizing the standard formula of prevalence as: 

n=z^2^p (1-p) 1d2

At 95% of confidential interval with acceptance sample error 6%. The sample was selected through non probability purposeful sampling technique. The inclusion criteria were only undergraduate physiotherapy students. Students who fulfills the criteria was included in the study after explaining study objectives and written informed consent was taken from participants. Postgraduates, Master, MPhil physiotherapy students and other medical and paramedical students were excluded from the study.

Depression, Anxiety and Stress were then scaled on the DASS (Depression, Anxiety and Stress Scale). The scale has been used and validated in several studies in the same population.^20^,^25^,^26^ It is designed to judge the three main psychological domains namely Depression, Anxiety and Stress. The 42 items of the questionnaire are cultural free and that make the test feasible to adapt to any culture.^18^ Physiotherapy students of either gender were invited to participate in the study. Data were entered and analyzed using SPSS version 21.0. Descriptive statistics including the frequency of depression, anxiety, stress and demographic characteristic of the participant were collected. All testing was applied at 95% confidence level. P-value is <0.05 was considered as significant.

## RESULTS

A total of 267 undergraduate physiotherapy students participated in the study. The survey response rate was 100%. Mean age of participants was 19.3±1.19 years. Around 201 (75.3%) students were female and 66 (24.7%) were male (Table-I). DASS-42 questionnaire was used to collect the frequency of depression, anxiety and stress. Data were interpreted as, Depression absent in 139 (52%) undergraduate physiotherapy students were as 44 (16.5%) involved mildly, 56 (21%) moderately, 23 (9%) severely and only 5 (2%) undergraduate physiotherapy students involved very severely. Anxiety was absent in around 84 (31.46%) undergraduate physiotherapy students out of 267. Those who had anxiety been categories as, students with mild anxiety were 37 (14%), moderate 64 (24%), severe 44 (16.5%) and very severe were 38 (14.2%). Similarly, stress was absent in 125 (46.82%) undergraduate physiotherapy students, whereas remaining were involved in it. Involved students categories according to DASS ranking criteria, those students who mildly involved in stress are 47 (17%), moderate are 66 (25%), severe are 24 (9%) and very severe are 5 (2%). shown in (Table-II). It is observed that Depression, anxiety and stress among undergraduates Physiotherapy students was 48%, 68.54% and 53.2%, respectively. They were calculated through adding all the categories like mild, moderate, severe and very severe of each of the variable (i.e. DASS).

**Table-I:** Characteristic of physiotherapy students of different institutes (n=267).

Sr.	Variable		n	%
1.	Age		19.3371(SD=1.18839)	
2.	Gender	Male	66	24.7
		Female	201	75.3
3.	Study Year	First Year	92	34.5
		Second Year	72	27.0
		Third Year	63	23.6
		Fourth Year	32	12.0
		Final Year	8	3.0

**Table-II:** Frequency of psychological morbidities using dass-42 (n=267).

Sr.	Tool	Category	Percentage (%)	Frequency(n)
1	DASS (Depression)	Normal	52.0	139
		Mild	16.479	44
		Moderate	20.97	56
		Severe	8.614	23
		Very Severe	1.872	5
2	DASS (Anxiety)	Normal	31.46	84
		Mild	13.857	37
		Moderate	23.97	64
		Severe	16.479	44
		Very Severe	14.232	38
3	DASS (Stress)	Normal	46.816	125
		Mild	17.602	47
		Moderate	24.72	66
		Severe	8.988	24
		Very Severe	1.8726	5

## DISCUSSION

The study shows that there is a considerable amount of depression, anxiety and stress among undergraduate physiotherapy students at various institutes in Sindh, Pakistan. This study is similar to other studies conducted earlier in Pakistan and in other countries. In contrast, present study was conducted on undergraduate physiotherapy students precisely.^2^,^4^,^12^-^21^

Depression in the past as well as in the present has been accredited with highest morbidity rate likewise anxiety and stress in medical education all over the globe.^2^,^26^ Thus it has been a subject of interest to the researchers. The present study highlights psychological morbidities including depression, anxiety and stress among physiotherapy students in Sindh, Pakistan. As well as the participants response was 100% thus validating the study results.

The prevalence of depression is 48.0%, anxiety 68.54% and stress 53.2% which is greater than that 24.4% of depression, 52% of anxiety and 16.9% of stress respectively among pre-clinical medical students reported by Faud et al.^18^

### Limitations of the study

The selection of study participants were limited to undergraduate students. The DASS score vary from institution to institution as there is no similarity in the academic calendar at time when data were collected. Additionally, there is no equal representation between years of study because there was unavailability of students due to semester breaks as well as due to examination in various institutes when study was conducted.

## CONCLUSION

More than half of the undergraduate physiotherapy students were found to be affected by depression, anxiety and stress. There is an urgent need to establish prevention programs and to bring out evidence based Psychological health promotion for Physiotherapy students in Pakistan. In order to help physiotherapy students to create smooth adjustments between different learning environments with changing learning needs and a growing academic burden.

### Authors’ Contributions

**AS:** Conceived, designed, did data collection, statistical analysis, interpretation and writing of manuscript.

**SS:** Did editing, critical revision and final approval of the manuscript.

**MK:** Conceived, designed and critical revision of manuscript.
